# Molecular identification of two thioredoxin genes and their function in antioxidant defense in *Arma chinensis* diapause

**DOI:** 10.3389/fphys.2024.1440531

**Published:** 2024-07-24

**Authors:** Zhongjian Shen, Qiaozhi Luo, Jianjun Mao, Yuyan Li, Mengqing Wang, Lisheng Zhang

**Affiliations:** ^1^ State Key Laboratory for Biology of Plant Diseases and Insect Pests, Key Laboratory of Natural Enemy Insects of Ministry of Agriculture and Rural Affairs, Institute of Plant Protection, Chinese Academy of Agricultural Sciences, Beijing, China; ^2^ School of Horticulture and Gardening, Tianjin Agricultural University, Tianjin, China; ^3^ Key Laboratory of Animal Biosafety Risk Prevention and Control (North) of Ministry of Agriculture and Rural Affairs, Shanghai Veterinary Research Institute, Chinese Academy of Agricultural Sciences, Shanghai, China

**Keywords:** *Arma chinensis*, thioredoxin, diapause, oxidative stress, antioxidant defense

## Abstract

Thioredoxin (Trx), an important part of thioredoxin systems, plays crucial role in maintaining the intracellular redox balance by scavenging reactive oxygen species (ROS). However, few Trxs have been functionally characterized in *Arma chinensis*, especially in diapause. In this study, diapause induction condition promoted hydrogen peroxide accumulation and increased CAT enzymatic activity and ascorbate content, suggesting that *A. chinensis* was exposed to high level of ROS. Therefore, we identified *AcTrx2* and *AcTrx-like*, and investigated the relationship with antioxidant defense. It was found that *AcTrx2* expression was significantly induced, whereas *AcTrx-like* expression was the highest on day 10 under diapause conditions. The expression of *AcTrx2* and *AcTrx-like* in fat body, a central metabolic organ of resisting oxidative stress, was significantly increased under diapause conditions, and was significantly improved by 5/15°C (diapause temperature). We investigated the knockdown of *AcTrx2* and *AcTrx-like* in *A. chinensis* and found that some selected antioxidant genes were upregulated, indicating that the upregulated genes may be functional compensation for *AcTrx2* and *AcTrx-like* silencing. We also found that the enzymatic activities of SOD and CAT, and the metabolite contents of hydrogen peroxide, ascorbate increased after *AcTrx2* and *AcTrx-like* knockdown. These results suggested the *AcTrx2* and *AcTrx-like* may play critical roles in antioxidant defense of *A. chinensis* diapause.

## 1 Introduction

Diapause is a developmental arrest stage in which insects respond to harsh environmental conditions, such as high summer temperatures, winter cold conditions, food limitation, or crowding (Guo et al., 2021a; Guo et al., 2021b). Lower temperature is usually used by insects to suppress their metabolism, conserve energy stores and gain cold resistance during winter diapause (Uzelac et al., 2024). However, lower temperatures can induce excessive reactive oxygen species (ROS), and high concentration of ROS has destructive effects on cells, including lipids, membranes, proteins, and nucleic acids (i.e., peroxide pressure) (Poli et al., 2004). In order to avoid the damage to cells caused by the toxicity of ROS, organisms have developed antioxidant enzymatic systems, including superoxide dismutase (SOD), thioredoxin peroxidase (TPx), peroxiredoxin (Prx) and thioredoxin (Trx) to maintain the intracellular ROS at proper levels (Kobayashi-Miura et al., 2007). Among these enzymes, Trx, a natural electron donor, is an important part of thioredoxin systems and plays essential roles in redox-regulatory processes (Kondo et al., 2006; Yoshioka et al., 2006; Lu and Holmgren, 2014).

Thioredoxin, a member of the small molecule reductase family, contains the typical disulfide bond active site Cys-Gly-Pro-Cys (CGPC), which is widely distributed from Archaea to human (Powis and Montfort, 2001; Lu and Holmgren, 2014). Since the first Trx is identified from *Escherichia coli* as a hydrogen donor for ribonucleotide reductase (Laurent et al., 1964), thioredoxin family genes have been identified and functionally studied in other living organisms, such as *Drosophila melanogaster* (Kanzok et al., 2001), *Spodoptera litura* (Kang et al., 2015), *Helicoverpa armigera* (Zhang et al., 2015), etc. Moreover, thioredoxin in mammals is mainly divided into two types, Trx1 and Trx2, which are located in the cytoplasm and mitochondria, respectively (Lu and Holmgren, 2014). Further studies have found that thioredoxin exerts antioxidant effects mainly through two aspects: one is as an electron donor of peroxidase, and the other is as a disulfide reductase of intracellular proteins (Kalinina et al., 2008).

Functionally, thioredoxin not only has the function of scavenging free radicals and protecting cells from oxidative stress (Chen et al., 2002; Revathy et al., 2012), but also has many physiological activities such as reducing hydrogen peroxide and regulating growth (Damdimopoulos et al., 2002). Studies on thioredoxin protecting organisms from ROS damage have focused on mammals, plants and bacteria (Benitez-Alfonso et al., 2009; Serata et al., 2012; Lee et al., 2013), but have been less studied in insects. In *D. melanogaster*, *Trx1*, *Trx2*, and *TrxT* have been cloned and identified (Bauer et al., 2002; Svensson et al., 2003), of which *Trx2* has been shown to be involved in regulating redox response and modulating the lifespan of flies (Bauer et al., 2002; Svensson and Larsson, 2007). Similarly, in *Apis cerana*, three *Trx* genes (*AccTrx-like1*, *AccTrx1*, and *AccTrx2*) have been demonstrated to play an important role in antioxidant defense (Lu et al., 2012; Yao et al., 2013; Yao et al., 2014). *HaTrx2*, in *H. armigera*, can reduce ROS production and larvae death caused by nucleopolyhedrovirus (NPV) (Zhang et al., 2015), while *GmTrx2* and *GmTrx-like1* from *Grapholita molesta* can resist oxidative stress caused by emamectin benzoate (Shen et al., 2018). Although the functional researches of *Trx* have been extended from model insects such as *D. melanogaster* to lepidoptera insects, there are few reports about its role in insect diapause.

*Arma chinensis* is the dominant natural enemy of many agricultural and forestry pests, and has strong predation ability on more than 40 kinds of pests, such as Lepidoptera, Coleoptera, Hemiptera and Hymenoptera (Meng et al., 2022). Unlike the photoperiod induced diapause of *Colaphellus bowringi* (Guo et al., 2021a), the diapause of *A. chinensis* dependes on low temperature. According to previous studies, characteristic active-site motifs of Trx are highly conserved and involved resistance to low-temperature induced oxidative stress in mammals and other species (Lu and Holmgren, 2014). Therefore, we predicted that *Trxs* may play an important role in diapause. In this study, we examined antioxidant enzyme activities and metabolite amounts of *A. chinensis* under diapause conditions, and have identified *AcTrx2* and *AcTrx-like*. Moreover, we further examined the temporal and spatial expression patterns of *AcTrx2* and *AcTrx-like* under diapause and non-diapause conditions and the differences in expression at low temperatures. Finally, we used RNA interference (RNAi) to detect the function of *AcTrx2* and *AcTrx-like* in resisting oxidative stress by detecting antioxidant enzyme activities, metabolite amounts, and the expression levels of other antioxidant genes. Our results provide a new perspective on the resistance mechanisms of *AcTrx2* and *AcTrx-like* to oxidative stress in *A. chinensis* diapause and help to develop strategies to improve survival during diapause of natural enemy insects.

## 2 Materials and methods

### 2.1 Insects

*Arma chinensis* were reared in our laboratory with *Antheraea pernyi* pupa at a constant temperature of 26°C± 1°C, under 70 %± 5% RH and a 16L: 8 D photoperiod. Diapause condition: the female adults within 24 h of emergence were placed at light ratio of 8L:16 D with transitions of temperatures 5/15°C, 70% ± 5% RH.

### 2.2 Difference of redox levels of *A*. *chinensis* under non-diapause or diapause condition

To detect oxidation and antioxidant levels in *A. chinensis* under two conditions, enzymatic capacity of superoxide dismutase (SOD) and catalase (CAT), and metabolite content of hydrogen peroxide (H_2_O_2_), and ascorbate were measured. Samples were collected from adults reared under diapause conditions for 10 days or non-diapause conditions for 10 days. The BCA Protein Assay Kit (Nanjing Jiancheng Bioengineering Institute, Nanjing, China) were used to extract and quantify the protein. According to the instructions, the catalase test kit, superoxide dismutase test kit, hydrogen peroxide test kit, ascorbate test kit (Nanjing Jiancheng Bioengineering Institute, Nanjing, China) were used to assay SOD enzymatic capacity, CAT enzymatic capacity, H_2_O_2_ content, and ascorbate content, respectively.

### 2.3 Gene identification and sequence analysis

The thioredoxin sequences were derived from previous transcriptome data of *Arma chinensi*s in our laboratory. The open reading frames (ORFs) of thioredoxin 2 (*Trx2*, PP820916) and thioredoxin-like (*Trx-like*, PP820915) were amplified using special primers (Supplementary Table S1) and then cloned into the pMD18-T vector (Takara Bio, Otsu, Japan) for sequencing (TSINGKE Bio, Beijing, China). Homologous protein sequences of *AcTrx2* and *AcTrx-like* from other species were obtained from National Center for Biotechnology Information (NCBI) (Supplementary Tables S2, S3), and analyzed using DNAman 6.0.3 software. Conserved domains in *AcTrx2* and *AcTrx-like* were predicted by NCBI services. Finally, MEGA v6.0 software was used to perform the phylogenetic analysis with the neighbor-joining method (1,000 bootstrap replicates).

### 2.4 Assays of RT-qPCR for temporal and spatial expression patterns

To detect the difference of temporal and spatial expression of *AcTrx2* and *AcTrx-like* in diapause condition and non-diapause condition, adults reared for 0, 10, 20, 30, 40 days under diapause conditions and adults reared for 0, 3, 6, 9, 12 days under non-diapause conditions were collected, respectively. Three biological replicates were taken at each time point and each treatment contained five adults. Samples of different tissues were collected from adults reared under diapause conditions for 10 days or non-diapause conditions for 10 days, including head, fat body, midgut and ovary. Each sample was repeated 3 times and each tissue was dissected from at least eight adults.

Total RNA was extracted by using TRIzol reagent (Takara, Kyoto, Japan), and the first-strand of cDNA was synthesized with PrimeScript RT reagent kit with gDNA Eraser (Takara, Kyoto, Japan). According to the manufacturer’s instructions, RT-qPCR was performed on LightCycler^®^ 96 Instrument (Roche (China) Holding Ltd., Shanghai, China) using cDNA and TB Green™ Premix Ex Taq™ (Takara, Kyoto, Japan) in a 20 µL reaction system. The primers used for RT-qPCR in this study were shown in Supplementary Table S1, and RPL27 gene was selected as endogenous reference gene. The results of qPCR were analyzed using the 2^−ΔΔCT^ method (Livak and Schmittgen, 2001).

### 2.5 Effect of temperature on *AcTrx2* and *AcTrx-like* expression

To determine the role of *AcTrx2* and *AcTrx-like* in resistance to low temperature during diapause, we examined the expression of genes at 5°C, 15°C and 5/15°C (diapause temperature). Samples were collected from adults exposed to 5°C, 15°C and 5/15°C for 24, 48, and 72 h, and the adults exposed to 26°C were as controls. Each treatment contained three biological replicates and each replicate contained six adults. All samples were immediately stored at −80°C for total RNA extraction, cDNA synthesis and qPCR detection.

### 2.6 dsRNA synthesis and injection of *AcTrx2* and *AcTrx-like*

To detect the function of *AcTrx2* and *AcTrx-like*, the gene expression levels were knocked down using RNA interference (RNAi). Double-stranded RNAs (dsRNAs) of *GFP*, *AcTrx2,* and *AcTrx-like* were synthesized by using MEGAscript RNAi kit (Ambion, California, United States) and the purified target sequences, which were amplified by RT-PCR using cDNA and special primers containing T7 polymerase promoter sequence (Supplementary Table S1). The NanoDrop™ 2000 spectrophotometer and agarose gel electrophoresis were used to detect the concentration and quality of dsRNA.

For RNAi of *AcTrx2* and *AcTrx-like*, 2-day postemergence adults were injected with dsRNA from each gene (2 μg/individual) by using a microinjector from the second and third abdominal segments. Samples were collected at 48 h after dsRNA injection. Adults were injected with dsGFP as a negative control. Biological replicates were performed 3 times in each treatment with at least 10 adults in each replicate. All samples were used for total RNA extraction, cDNA synthesis and RT-qPCR detection to examining gene interference efficiency.

### 2.7 Transcriptional levels of other antioxidant genes after knockdown of *AcTrx2* and *AcTrx-like*

After *AcTrx2* and *AcTrx-like* knockdown, three other antioxidant genes, including glutaredoxin 5 (*Grx5*), glutaredoxin domain-containing cysteine-rich protein (*GrxCR*), protein disulfide isomerase (*PDI*) were examined. RT-qPCR was used to detect the expression levels of *Trx-like*, *GrxCR*, *Grx5*, and *PDI* after *AcTrx2* knockdown. Expression levels of *Trx*, *GrxCR*, *Grx5*, and *PDI* were determined following *AcTrx-like* silencing.

### 2.8 Analysis of enzymatic activity and metabolite content after knockdown of *AcTrx2* and *AcTrx-like*

To determine the role of *AcTrx2* and *AcTrx-like* in antioxidant function, we examined the enzymatic activities of superoxide dismutase (SOD) and catalase (CAT) and metabolite content of H_2_O_2_, and ascorbate after genes knockdown. Protein samples were collected at 48 h after dsRNA injection and were extracted and quantified with the BCA Protein Assay Kit (Nanjing Jiancheng Bioengineering Institute, Nanjing, China). Then, according to the instructions, a catalase test kit and a superoxide dismutase test kit (Nanjing Jiancheng Bioengineering Institute, Nanjing, China) were used to assay enzymatic capacity of SOD and CAT, respectively. Similarly, the metabolite content of H_2_O_2_, and ascorbate were quantified using hydrogen peroxide, ascorbate test kit (Nanjing Jiancheng Bioengineering Institute, Nanjing, China), respectively, referring to the instructions.

### 2.9 Statistical analysis

The SPSS 21 software was used to perform statistical analysis of experimental datas and values were represented as the means ± stand error of mean (SEM) with at least three independent repetitions. Gene expression in different tissues were analyzed using ANOVA, followed by a Turkey’s HSD multiple comparison test, with a and b indicating significant differences at *p* < 0.05. Other RT-qPCR results and the results of enzymatic activity and metabolite content were analyzed by using the pair-wise Student’s *t*-test, and significance levels were denoted by * (0.01 < *p* < 0.05) and ** (*p* < 0.01).

## 3 Results

### 3.1 Difference of oxidation level of *A. chinensis* between non-diapause and dipause conditions

**FIGURE 1:**
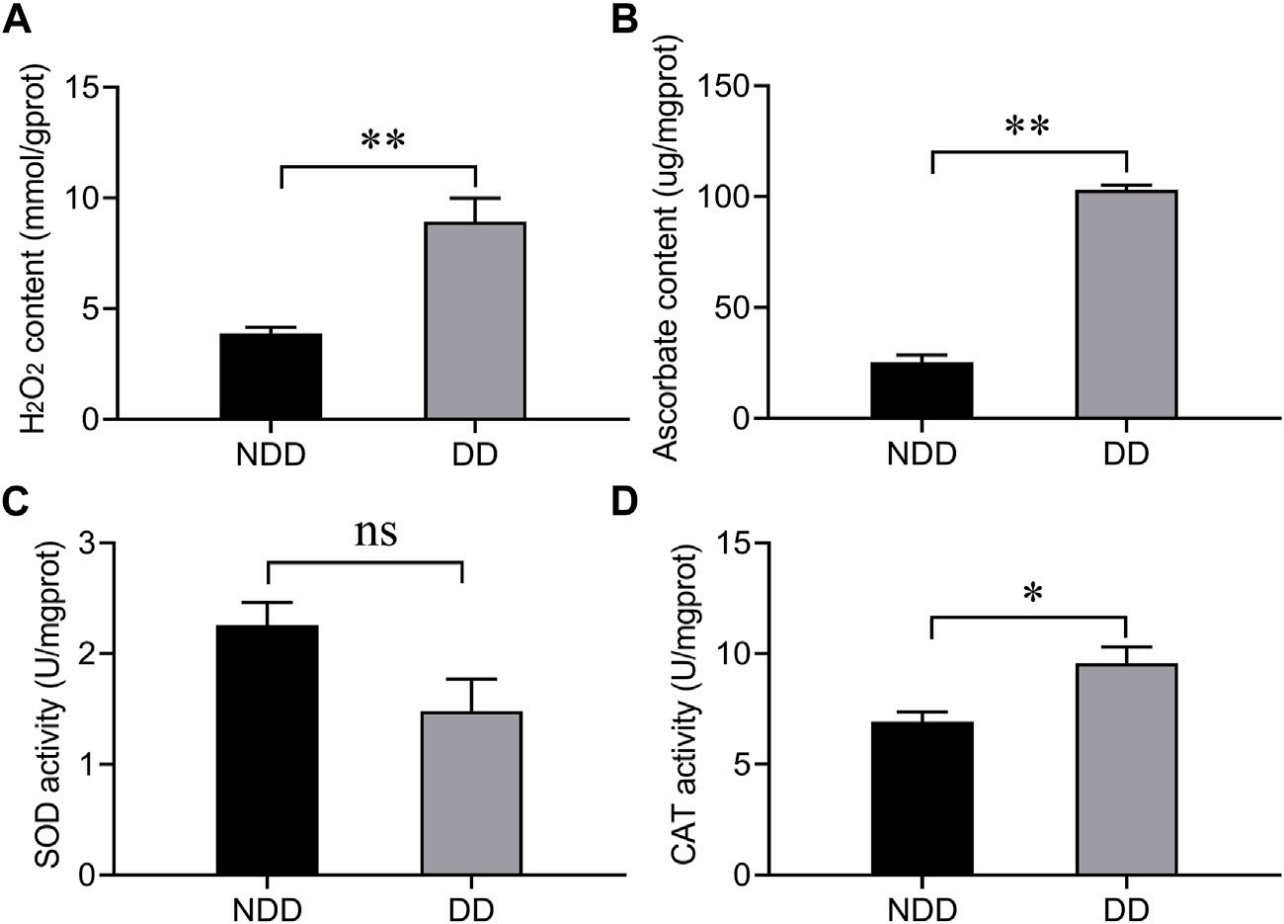
Determination of **(A)** hydrogen peroxide, **(B)** ascorbate, and the enzymatic activity of antioxidants **(C)** SOD, and **(D)** CAT under no-diapause and diapause conditions. Adults were collected at 10 days after emergence under no-diapause and diapause conditions, respectively. NDD: non-diapause-destined female refers to the adults were raised for 10 days in reproduction conditions; DD: diapause-destined female refers to the adults were raised for 10 days in diapause conditions. Each value represents the mean ± stand error of mean (SEM). Asterisks indicate significant differences between NDD and DD using Student’s *t*-test (*, 0.01 < *p* < 0.05; **, *p* < 0.01).

To determine the oxidation level of *A. chinensis* under diapause conditions, the content of hydrogen peroxide (H_2_O_2)_ and ascorbate, and the enzymatic activities of superoxide dismutase (SOD) and catalase (CAT) were examined. As shown in Figure 1, compared with no-diapause group, the H_2_O_2_ and ascorbate contents were significantly increased (Figures 1A,B), and the enzymatic activity of CAT was also significantly enhanced (Figure 1D), but there was no difference in SOD capacity (Figure 1C). The above results suggested that *A. chinensis* was exposed to ROS under diapause condition.

### 3.2 Cloning and sequence analysis of *AcTrx2* and *AcTrx-like*

**FIGURE 2:**
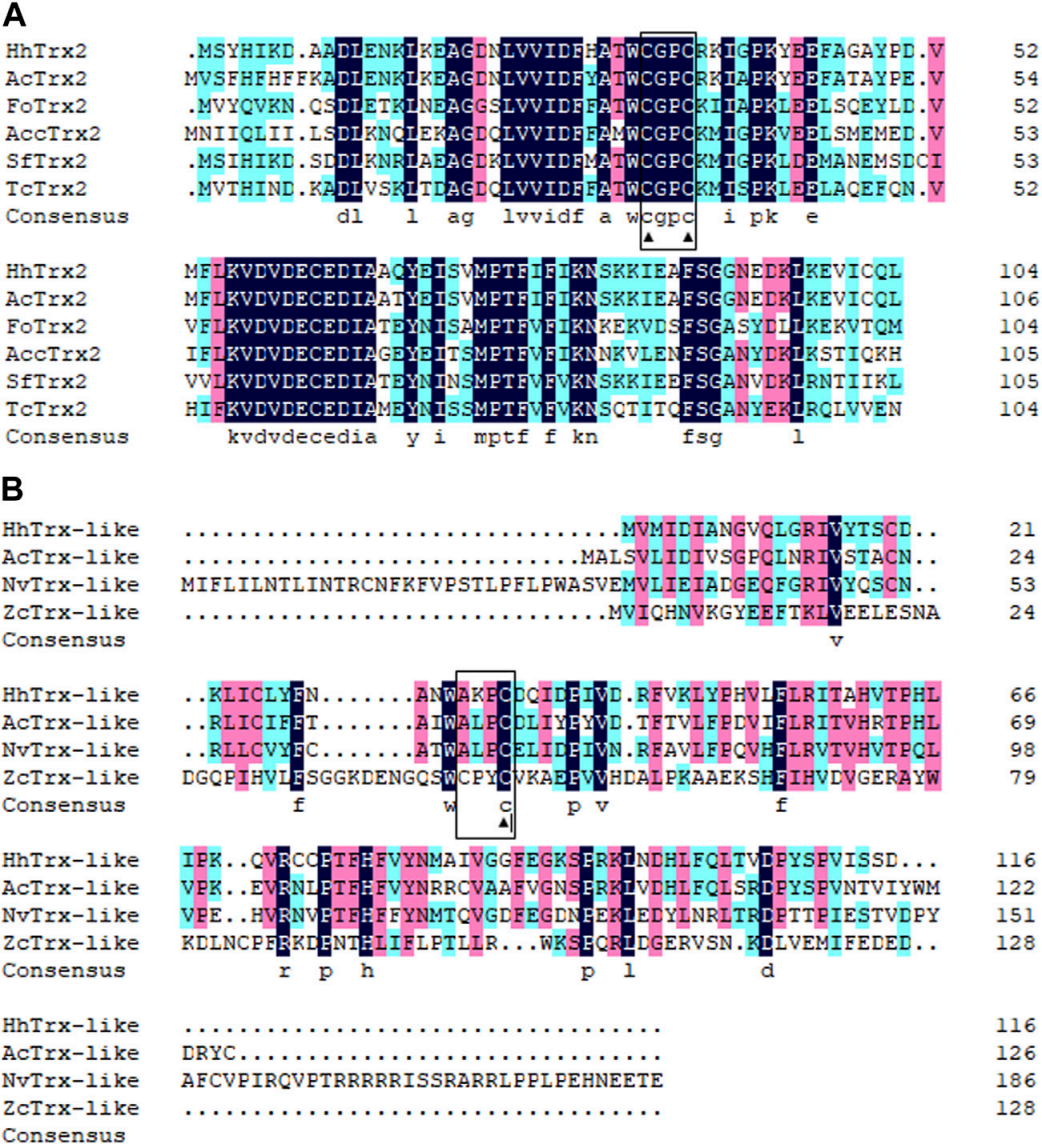
Multiple alignments of **(A)** *AcTrx2* and **(B)** *AcTrx-like* with homologs from other insect species. The conserved CGPC motif and ALPC motif were boxed and the active sites were marked by ▲. The protein names and accession numbers were listed in Supplementary Table S2.

**FIGURE 3:**
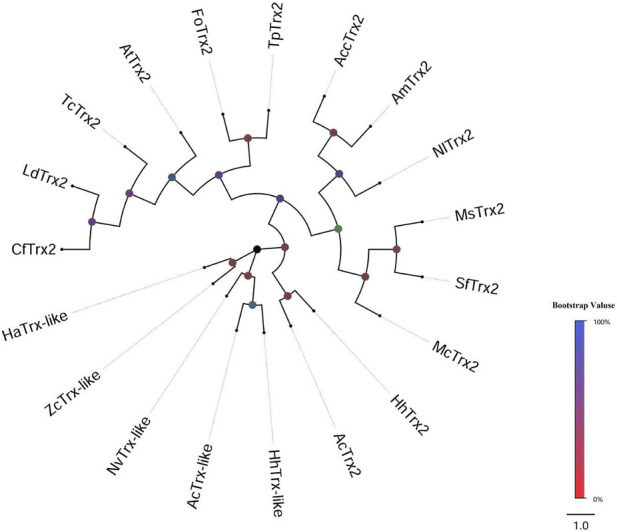
Phylogenetic analysis of *AcTrx2* and *AcTrx-like* with homologs from other insect species. The phylogenetic tree was constructed based on amino acid sequences of *AcTrx2* and *AcTrx-like* using the MEGA-based neighbor-joining method with 1,000 bootstraps. The protein names and accession numbers were listed in Supplementary Table S3.

Two distinct cDNA fragments were identified from the transcriptome database of *A. chinensis*, and the two gene sequences were analyzed with different molecular characteristics using the NCBI-ORF Finder and NCBI-BLAST, subsequently named *AcTrx2* and *AcTrx-like*. Sequence analysis showed that the open reading frame (ORF) of *AcTrx2* and *AcTrx-like* was 324 bp and 381 bp, encoding 107 amino acids and 126 amino acids, respectively (Supplementary Figure S1, S2). Multiple sequence alignment revealed *AcTrx2* was 57%–88% sequence identity with homologous amino acid sequences from other species and possessed a highly conserved CGPC active-site motif (Figure 2A). Low amino acid identity (10%–58%) between *AcTrx-like* and other *Trx-like* sequences including *Halyomorpha halys*, *Nezara viridula*, and *Zeugodacus cucurbitae*, and an active site sequence ALPC was found in the *A. chinensis* (Figure 2B). Phylogenetic analysis revealed that *AcTrx2* was most closely related to the *HhTrx2* homologue (Figure 3A), and *AcTrx-like* was the most closely related to *HhTrx-like* than other selected species (Figure 3B). These results were consistent with the predictive evolutionary relationship of multiple alignments of amino acid sequences.

### 3.3 Spatio-temporal expression profiles of *AcTrx2* and *AcTrx-like*

**FIGURE 4:**
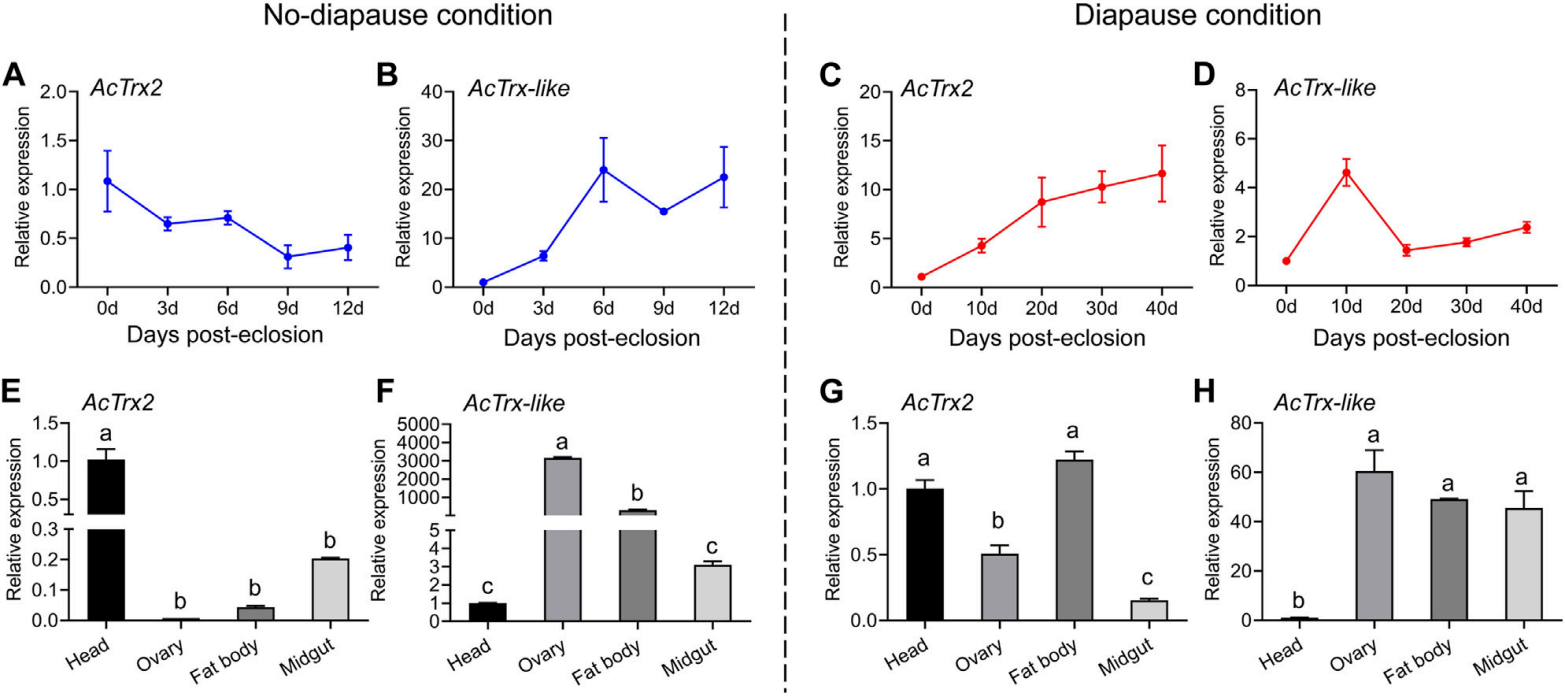
Expression patterns of *AcTrx2* and *AcTrx-like* at different developmental stages and in different tissues. **(A–D)** Temporal expression profiles of *AcTrx2* and *AcTrx-like* under non-diapause and diapause conditions. **(E–H)** Spatial transcript expression analysis of *AcTrx2* and *AcTrx-like* under non-diapause and diapause conditions. Data represent mean ± stand error of mean (SEM). Different letters indicated statistically significant differences between different tissues using a one-way analysis of variance (ANOVA) with Tukey’s multiple comparisons test, *p* < 0.05.

RT-qPCR was used to investigate the transcriptional expression of *AcTrx2* and *AcTrx-like* under non-diapause and diapause conditions at various developmental stages, and in different tissues of *A. chinensis*. The results of temporal expression profile showed that the expression of *AcTrx2*, in non-diapause conditions, decreased with the development time, and the highest expression appeared at 0 days of emergence (Figure 4A), while in diapause conditions, the expression of *AcTrx2* increased gradually with developmental time, and the lowest expression was at 0 days of emergence (Figure 4C). *AcTrx-like* was highly expressed in 6, 9, and 12-day postemergence adults in non-diapause conditions (Figure 4B). However, under diapause conditions, the expression levels of *AcTrx-like* showed an overall upward trend, and the highest expression was at 10 days after *A. chinensis* emergence (Figure 4D). For spatial expression, *AcTrx2* was the most highly expressed in head from *A. chinensis* adults in non-diapause conditions (Figure 4E), and the expression of *AcTrx2* in the head and fat body was the highest in diapause condition, followed by the ovary (Figure 4G). *AcTrx-like* transcriptional expression was the highest in the ovary and the lowest in the head under non-diapause conditions (Figure 4F), whereas in diapause conditions, *AcTrx-like* expression was highest in ovary, fat body and midgut (Figure 4H).

### 3.4 Expression profiles of *AcTrx2* and *AcTrx-like* at different temperatures

**FIGURE 5:**
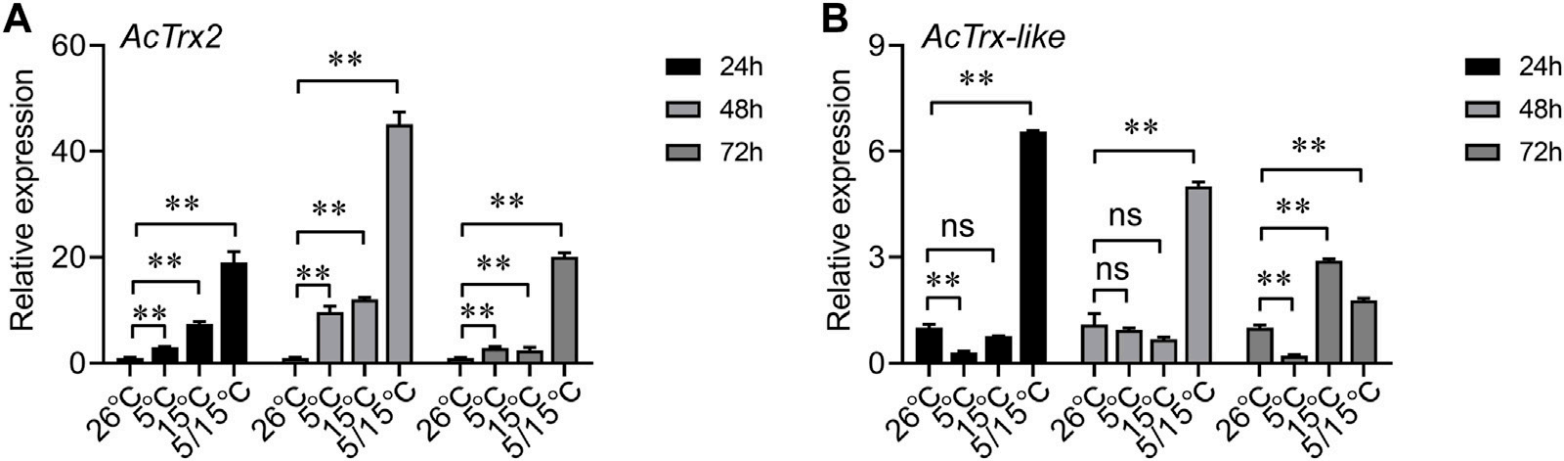
Expression patterns of **(A)** *AcTrx2* and **(B)** *AcTrx-like* in adults after exposure to different temperatures. Total RNA was harvested from adults of *A. chinensis* samples following temperature challenges, including 5°C, 15°C, and 5/15°C. Data represent mean ± stand error of mean (SEM). Asterisks indicate significant differences between different temperatures using Student’s *t*-test (*, 0.01 < *p* < 0.05; **, *p* < 0.01), ns: no significance.

To determine the effect of low temperature on *AcTrx2* and *AcTrx-like* expression, we examined genes expression at 5°C, 15°C, and 5/15°C. RT-qPCR results showed that *AcTrx2* expression was significantly induced after *A. chinensis* exposure to three low temperatures for 24, 48, and 72 h, and *AcTrx2* was most sensitive to the 5/15°C, which is the diapause temperature (Figure 5A). Under stress of 5/15°C, *AcTrx-like* was all upregulated at 24, 48, and 72 h, while at 15°C, *AcTrx-like* expression was induced only at 72 h after treatment (Figure 5B). In the 5°C treatment, the expression of *AcTrx-like* was significantly inhibited at 24 and 72 h, but there was no significant difference at 48 h (Figure 5B).

### 3.5 Expression profiles of other antioxidant genes after knockdown of *AcTrx2* and *AcTrx-like*

**FIGURE 6:**
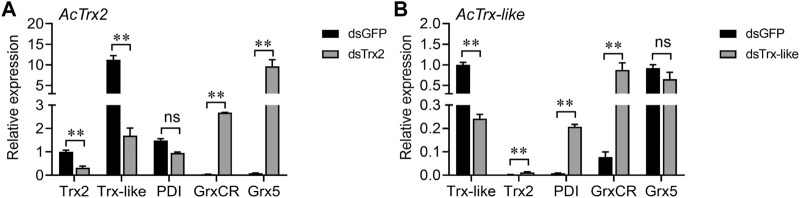
Expression analysis of other antioxidant genes after the knockdown of **(A)** *AcTrx2* and **(B)** *AcTrx-like*. The samples were collected at 48 h after dsRNA injection of *GFP*, *Trx2* and *Trx-like* and then subjected to total RNA extraction and qRT-PCR analysis. *Grx5*, glutaredoxin 5; *GrxCR*, glutaredoxin domain-containing cysteine-rich protein; *PDI*, protein disulfide isomerase. Data represent mean ± stand error of mean (SEM). Asterisks indicate significant differences between different temperatures using Student’s *t*-test (*, 0.01 < *p* < 0.05; **, *p* < 0.01), ns: no significance.

RT-qPCR was used to detect changes in the expression of other antioxidant genes after *AcTrx2* and *AcTrx-like* silencing to reveal the relationship between these two genes and other antioxidant genes. When the expression of *AcTrx2* decreased by 67.9%, the expression of *AcGrxCR* and *AcGrx5* increased significantly, while the expression of *AcTrx-like* decreased, and the expression of *AcPDI* did not change (Figure 6A). Compared to the GFP groups, *AcPDI* and *AcGrxCR* were upregulated after *AcTrx-like* suppressed by 75.9%, but *AcTrx2* expression was downregulated, and *AcGrx5* had no difference in expression (Figure 6B).

### 3.6 Effects of *AcTrx2* and *AcTrx-like* knockdown on antioxidant enzyme activities and metabolite contents

**FIGURE 7:**
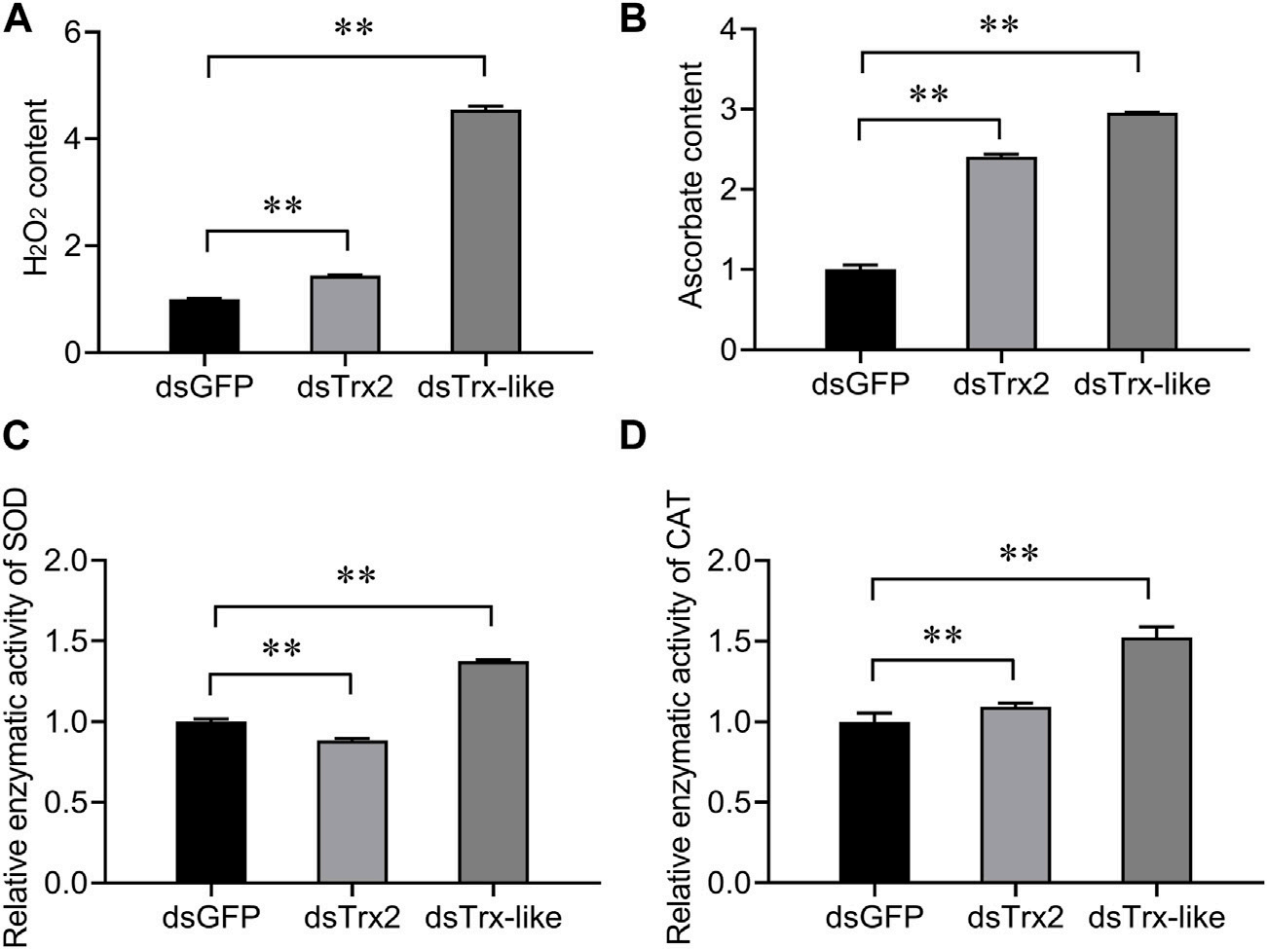
Effects of *AcTrx2* and *AcTrx-like* knockdown on metabolite contents of **(A)** hydrogen peroxide and **(B)** ascorbate, and enzymatic activity of **(C)** SOD and **(D)** CAT. Whole adults were collected at 48 h after *GFP*, *AcTrx2*, and *AcTrx-like* dsRNA injection and then analyzed using the corresponding kits according to the manufacturer’s protocol. Data represent mean ± stand error of mean (SEM). Asterisks indicate significant differences between different temperatures using Student’s *t*-test (*, 0.01 < *p* < 0.05; **, *p* < 0.01).

To elucidate the function of *AcTrx2* and *AcTrx-like* in antioxidant defense in *A. chinensis*, superoxide dismutase (SOD) capacity, catalase (CAT) capacity, hydrogen peroxide (H_2_O_2_) content, and ascorbate content of *A. chinensis* were examined. As shown in Figure 7, compared with the control group, when *AcTrx2* and *AcTrx-like* were silenced, the H_2_O_2_ and ascorbate contents were significantly increased (Figures 7A,B), and the enzymatic activity of CAT was also significantly enhanced (Figure 7D). However, SOD capacity decreased after *AcTrx2* was suppressed, and improved after *AcTrx-like* knockdown (Figure 7C). All the above results indicated that these two *Trx* genes may be involved in antioxidant defense in *A. chinensis*.

## 4 Discussion

Diapause is a characteristic of insects to avoid harsh environment, and it is an important mean to extend the shelf life of natural enemy products to realize commercialization (Denlinger, 2008). Large number of previous studies mainly focused on the mechanism of diapause regulation, such as the role of juvenile hormone (JH), insulin, etc., in diapause (Tian et al., 2021; Chen et al., 2024). However, there are few studies on insect resistance to low temperature, which is usually used to induce insect winter diapause and causes an increase in reactive oxygen species (ROS) leading to oxidative damage (El-Saadi et al., 2023). In this study, we found that the content of hydrogen peroxide and ascorbate, and the enzymatic activity of CAT increased in diapause condition, indicating that *A. chinensis* was exposed to ROS and used antioxidant enzymes and antioxidant substances to resist oxidative damage. In addition to these, there may be other antioxidant genes involved in eliminating ROS. As far as we know, Trxs can maintain cellular redox homeostasis and participate in resistance to oxidative damage caused by ROS (Holmgren, 1985; Arnér and Holmgren, 2000; Myers et al., 2008). Hence, we hypothesized that *Trxs* regulates oxidation levels during diapause induction of *A. chinensis*.

In previous studies, *AcTrx2* and *AcTrx-like* were identified and elucidated their role in the reproductive diapause of *A. chinensis*. *AcTrx2* had high amino acid identity (57%–88%) with other insect counterparts, and contained highly conserved active sites motif-CGPC. The highly conserved redox-active dithiol group in the form of CXXC is essential for catalytic activity of Trxs (Kallis and Holmgren, 1980; Powis and Montfort, 2001). *AcTrx-like* sequence contained ALPC-active site sequence. These results demonstrated that *AcTrx2* and *AcTrx-like* possessed cysteine residues of redox active sites to maintain the intracellular redox balance. *AcTrx2* gene was expressed at higher levels in the head, in accordance with other insects, including *H. armigera* (Zhang et al., 2015), *A. cerana* (Yao et al., 2013), while *AcTrx-like* was highest expressed in the ovaries. Temporal expression profiles showed that the expression of *AcTrx2* was lower in non-diapause conditions, but significantly increased in diapause conditions, while *AcTrx-like* was highly expressed in non-diapause conditions at the spawning stage, and the expression was the highest at 10 days under diapause conditions. Moreover, the expressions of *AcTrx2* and *AcTrx-like* in fat body, which is the central metabolic organ that plays an important role in detoxifification/degradation of xenobiotics and resisting oxidative stress (Enayati et al., 2005; Arrese and Soulages, 2010), were significantly improved under diapause conditions. Therefore, we speculated that *AcTrx2* and *AcTrx-like* played an important role in diapause.

Previous studies have shown that many environmental factors, including abnormal temperatures, can cause oxidative stress (Lushchak, 2011). Low temperature has been shown to induce *Trxs* expression in insects, such as *H. armigera* (Zhang et al., 2015), *G. molesta* (Shen et al., 2018), and *A. cerana* (Yao et al., 2013; 2014). In the present study, *AcTrx2* expression was significantly induced by low temperature (5°C, 15°C, 5/15°C), especially diapause temperature of 5/15°C. Similarly, *AcTrx-like* transcription levels were also significantly increased in 5/15°C, although its expression was suppressed in 5°C. These results further demonstrated that both *AcTrx2* and *AcTrx-like* were involved in resistance to oxidative stress induced by low-temperature during diapause.

High levels of ROS lead to DNA damage, lipid peroxidation, formation of protein disulfide bond, and protein carbonylation (Kalinina et al., 2014). Antioxidant enzymes such as CAT, SOD and antioxidant substances (ascorbate, total GSH) are used by cells to remove excessive ROS and resist oxidative damage (Meister, 1994; Corona and Robinson, 2006). In *Drosophila,* the loss of *Trx2* promotes the expression of other antioxidant genes and exacerbates oxidative stress-dependent phenotypes (Tsuda et al., 2021). In *A. cerana*, the enzymatic activities of CAT and POD, and the metabolite content of H_2_O_2_, carbonyls, and ascorbate were increased after *AccTrx1* was knocked down, and the expression levels of *AccCAT*, *AccGSTS4*, *AccMsrA*, and *AccTrx2* were elevated (Yao et al., 2014). In this study, after *AcTrx2* and *AcTrx-like* knockdown, H_2_O_2_ content, ascorbate content and CAT capacity were significantly increased, and the enzymatic activity of SOD was also enhanced after *AcTrx-like* silencing, revealing that *A. chinensis* was exposed to oxidative stress, and CAT, SOD, and ascorbate may be involved in scavenging ROS. In addition, *AcGrxCR* and *AcGrx5* expression levels were enhanced after *AcTrx2* knockdown, while *AcPDI* and *AcGrxCR* expression levels were significantly induced when *AcTrx-like* was silenced, revealing that all above induced genes were involved in compensating for the knockdown of *AcTrx2* and *AcTrx-like* to resist oxidative damage.

In conclusion, the diapause induced process of *A. chinensis* increased reactive oxygen species content and altered antioxidant capacity. Moreover, *AcTrx2* and *AcTrx-like* from *A. chinensis* were successfully identified and possessed conserved functional domains of the Trx superfamilies. The expression levels of *AcTrx2* and *AcTrx-like* were significantly improved under diapause conditions and induced by low temperature, especially 5/15°C, implying that *AcTrx2* and *AcTrx-like* may play critical roles in scavenging excess ROS and in antioxidant defense. After RNAi mediated knockdown of *AcTrx2* and *AcTrx-like*, the expression levels of some other antioxidant genes (such as *GrxCR*, *Grx5*, etc.) were upregulated, the enzymatic activities of SOD and CAT increased, and metabolites (hydrogen peroxide and ascorbate) also increased. Our study verified the antioxidant roles of *AcTrx2* and *AcTrx-like* in *A. chinensis* diapause, providing new insights into the mechanism of diapause in insects.

## Data Availability

The datasets presented in this study can be found in online repositories. The names of the repository/repositories and accession number(s) can be found in the article/Supplementary Material.
