# Supplementary material for: Molecular identification of two thioredoxin genes and their function in antioxidant defense in Arma chinensis diapause
Source: Front Physiol. 2024 Jul 24;15:1440531. doi: 10.3389/fphys.2024.1440531 (PMC11303210; doi:10.3389/fphys.2024.1440531)
Supplement: Supplementary file 1 [file DataSheet1.docx]

Supplementary Material

Molecular identification of two thioredoxin genes and their function in antioxidant defense in *Arma chinensis* diapause

Zhongjian Shen, Qiaozhi Luo, Jianjun Mao, Yuyan Li, Mengqing Wang and Lisheng Zhang*

*** Correspondence:** Lisheng Zhang: zhangleesheng@163.com

# Supplementary Figures and Tables

## Supplementary Figures


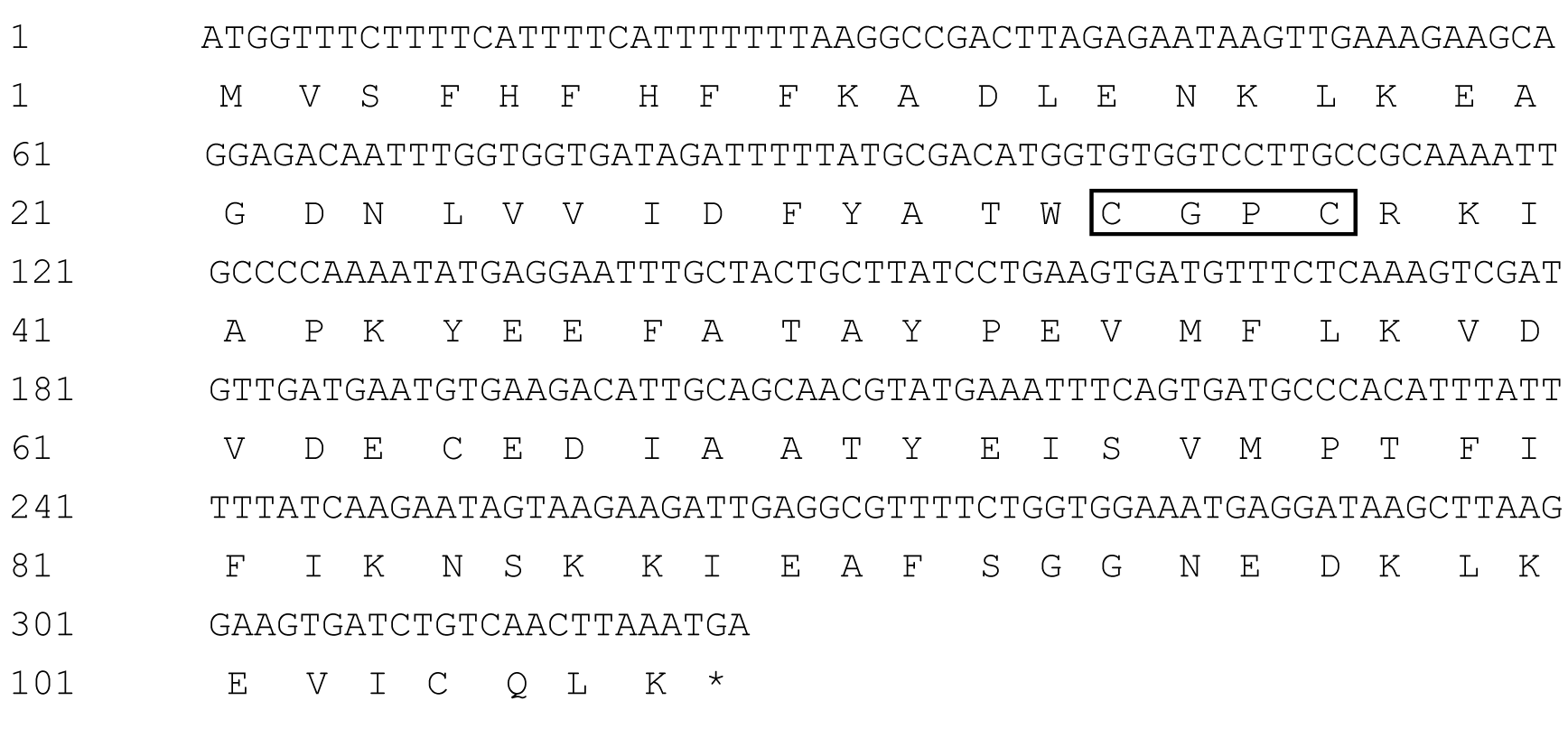


**Supplementary Figure S1. Nucleotide and deduced amino acid sequences of *AcTrx2* gene from *A. chinensis***

*
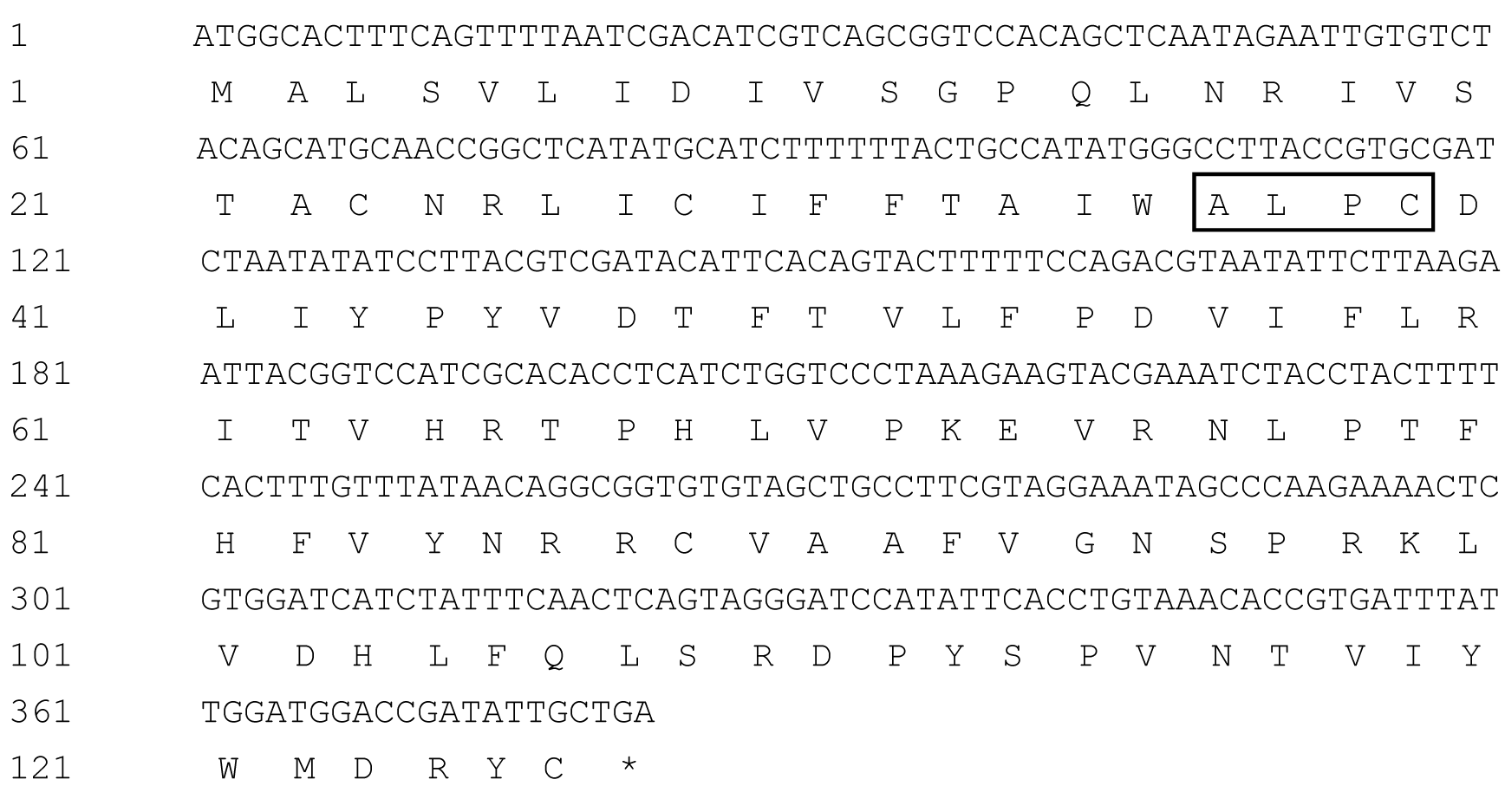
*

**Supplementary Figure S2. Nucleotide and deduced amino acid sequences of *AcTrx-like* gene from *A. chinensis***

# 1.2 Supplementary Tables

**Table S1: Primers used in this study**

| **Gene** | **Direction** | **Sequence (5′-3′)** |
| --- | --- | --- |
| **cDNA cloning** | | |
| *AcTrx2* | F | TTGCTTTGTGGTTAGTCAGTTG |
|  | R | GTTCTGCATTAAACTTCCAAGC |
| *AcTrx-like* | F | ACTACGATTTGTCGCACCG |
|  | R | TGACCAGGACGAATAAGACATA |
| **RT-qPCR** | | |
| *AcTrx2* | F | ATGGTTTCTTTTCATTTTC |
|  | R | AATTTTGCGGCAAGGACC |
| *AcTrx-like* | F | ATGGCACTTTCAGTTTTAATCG |
|  | R | GCATATGAGCCGGTTGCAT |
| *AcGrxCR* | F | ATGGACCAGAGATCTTTTAC |
|  | R | CTGCTCGCTGTCACTACAAGT |
| *AcGrx5* | F | ATGAATTATTTGATTAGGTC |
|  | R | CCGCAGCACTTGAAAGTAGTCT |
| *AcPDI-F* | F | TCAAGGTGACAGGACAAAGG |
|  | R | TTTCTTGGCGTGTTATTTGC |
| *AcRPL27* | F | CCACCTTAGACGTAACCAGAA |
|  | R | ACAGTCGATAACTGGTGCCT |
| **RNA interference** | | |
| *dsRNA-Trx2* | F | TAATACGACTCACTATAGGGGGTCCTTGCCGCAAAATT |
|  | R | TAATACGACTCACTATAGGGTCATTTAAGTTGACAGATCAC |
| *dsRNA-Trx-like* | F | TAATACGACTCACTATAGGGATGCAACCGGCTCATATGC |
|  | R | TAATACGACTCACTATAGGGTCAGCAATATCGGTCCATCCA |
| *dsRNA-GFP* | F | TAATACGACTCACTATAGGGCACAAGTTCAGCGTGTCCG |
|  | R | TAATACGACTCACTATAGGGAGTTCACCTTGATGCCGTTC |

**Table S2 GenBank accession numbers of other species homologous sequences used for the multiple sequence alignment.**

| **Species name** | **Protein** | **Acc. number** |
| --- | --- | --- |
| *Halyomorpha halys* | thioredoxin-2 | XP_014271868.1 |
| *Frankliniella occidentalis* | thioredoxin-2 | XP_026273895.1 |
| *Apis cerana cerana* | thioredoxin-2 | PBC30074.1 |
| *Spodoptera frugiperda* | thioredoxin-2 | XP_035450902.1 |
| *Tribolium castaneum* | thioredoxin-2 | XP_967987.1 |
| *Halyomorpha halys* | thioredoxin-like | XP_014286559.1 |
| *Nezara viridula* | thioredoxin-like | CAH1397241.1 |
| *Helicoverpa armigera* | thioredoxin-like | AKS26495.1 |
| *Zeugodacus cucurbitae* | thioredoxin-like | XP_011192212.1 |

**Table S3 GenBank accession numbers of other species used for phylogenetic analysis.**

| **Species name** | **Protein** | **Acc. number** |
| --- | --- | --- |
| *Halyomorpha halys* | thioredoxin-2 | XP_014271868.1 |
| *Frankliniella occidentalis* | thioredoxin-2 | XP_026273895.1 |
| *Thrips palmi* | thioredoxin-2 | XP_034254050.1 |
| *Apis cerana cerana* | thioredoxin-2 | PBC30074.1 |
| *Aethina tumida* | thioredoxin-2 | >XP_019875278.1 |
| *Manduca sexta* | thioredoxin-2 | XP_030041213.1 |
| *Cylas formicarius* | thioredoxin-2 | XP_060533926.1 |
| *Spodoptera frugiperda* | thioredoxin-2 | XP_035450902.1 |
| *Leptinotarsa decemlineata* | thioredoxin-2 | XP_023014571.1 |
| *Neodiprion lecontei* | thioredoxin-2 | XP_015510550.1 |
| *Apis mellifera* | thioredoxin-2 | XP_003250408.1 |
| *Melitaea cinxia* | thioredoxin-2 | XP_045457450.1 |
| *Tribolium castaneum* | thioredoxin-2 | XP_967987.1 |
| *Halyomorpha halys* | thioredoxin-like | XP_014286559.1 |
| *Nezara viridula* | thioredoxin-like | CAH1397241.1 |
| *Helicoverpa armigera* | thioredoxin-like | AKS26495.1 |
| *Zeugodacus cucurbitae* | thioredoxin-like | XP_011192212.1 |
